# Adverse and positive childhood experiences in relation to adolescent mental health: sequential indirect associations

**DOI:** 10.3389/fpsyg.2026.1799835

**Published:** 2026-06-18

**Authors:** Yulong Li, Yanting Cao, Lingying Liao, Qing Li, Xiaoli Chen

**Affiliations:** Faculty of Teacher Education, Baise University, Baise, Guangxi, China

**Keywords:** adverse childhood experiences, mental health, positive childhood experiences, psychological resilience, rumination

## Abstract

**Background:**

Accumulating evidence has linked adverse childhood experiences (ACEs) to adolescent mental health problems, whereas positive childhood experiences (PCEs) may serve a protective function. Guided by the stress-vulnerability model, this study examined the associations between ACEs and adolescent mental health, and further investigated the indirect pathways via rumination and psychological resilience, as well as the moderating role of PCEs.

**Methods:**

A total of 700 adolescents were recruited and completed validated measures of ACEs, PCEs, rumination, psychological resilience, and mental health. Indirect association and moderation models were tested using the PROCESS macro in SPSS, and the significance of indirect associations was evaluated with bootstrap confidence intervals.

**Results:**

ACEs were significantly associated with poorer mental health. Rumination and psychological resilience each showed significant indirect associations, and together formed a significant sequential indirect association in the cross-sectional model. In addition, PCEs significantly moderated the associations between ACEs and mental health, between ACEs and rumination, and between ACEs and psychological resilience.

**Conclusion:**

These findings suggest a complex pattern of associations between ACEs and adolescent mental health: rumination and psychological resilience may be involved in a sequential indirect association, and PCEs were associated with a protective moderating pattern. The results may inform risk identification for adolescent mental health and the development of family- and school-based intervention strategies.

## Introduction

Mental health (MH) serves as a crucial foundation for the overall development of adolescents, encompassing their abilities in emotional regulation, interpersonal communication, stress coping, and behavioral control (Xu et al., 2025). A sound state of MH not only fosters positive self-identity, enhances learning motivation, and promotes social adaptability, but also contributes to improved social functioning and life satisfaction (Carlén et al., 2023). Adolescents often undergo significant physical and psychological developments while simultaneously facing multiple stressors related to academics, peer relationships, and future uncertainties. These pressures make them particularly susceptible to MH problems, which can have far-reaching consequences (Zhou et al., 2023). It is estimated that approximately 14% of children and adolescents worldwide suffer from MH disorders, making this issue a major public health concern (World Health Organization, 2022). In China, more than 30 million children and adolescents are reported to be dealing with emotional or behavioral difficulties (National, Health, and Wellness Commission of the People's Republic of China, 2021). Empirical research has demonstrated that adolescents with better MH are more capable of managing external challenges, maintaining emotional stability, and exercising behavioral discipline. As a result, they tend to perform better across academic, familial, and social settings (Göbel et al., 2022). Conversely, poor MH is linked to a heightened risk of depression, anxiety, and self-harm, which can disrupt normal development and increase the likelihood of psychological disorders in adulthood (Wang et al., 2025). The results emphasize the critical of prioritizing adolescent MH as a central concern in psychological research and educational practice.

Adolescent MH is closely linked to the interplay between early life experiences and individual psychological traits (Bakir and Dilmaç, 2025; Eriksson and Stattin, 2023). Childhood represents a critical period for shaping emotional regulation, security, and coping capacities, and the quality of early environments has profound implications for subsequent psychological adaptation (Hou et al., 2022). In particular, adverse childhood experiences (ACEs) such as neglect, abuse, and emotional deprivation are consistently associated with a heightened likelihood of psychological conditions (Gajos et al., 2022; Song et al., 2024). However, the underlying cognitive and emotional processes that may help explain these associations remain insufficiently understood. Recent studies have suggested that rumination, a maladaptive form of cognitive processing, may function as a putative mediating variable linking ACEs to MH outcomes (Masuya et al., 2023). Persistent engagement in repetitive negative thinking may be associated with intensified emotional distress and limited the mobilization of internal psychological resources. In contrast, psychological resilience (PR), widely regarded as a core protective factor in managing stress, has also been implicated in the association between early-life risk and subsequent MH (Chen et al., 2023). Moreover, emotional support and positive reinforcement received during early developmental stages may be linked to greater psychological security and adaptability, thereby attenuating the negative associations between ACEs and MH (Van Doorn et al., 2024). While previous research has primarily concentrated on the direct association between ACEs and MH or considered isolated putative mediators, few have explored the sequential statistical indirect associations involving cognitive and emotional mechanisms in an integrated framework. In addition, the potential moderating role of positive childhood experiences (PCEs) has been largely overlooked, leaving the protective functions of early positive environments underexplored.

To systematically examine how childhood experiences are associated with adolescent MH, the stress-vulnerability model (SVM) was used as the theoretical framework (Zubin and Spring, 1977). This model posits that psychological distress is related to the interaction between internal vulnerabilities and external stressors. When individuals are exposed to adverse early-life environments, a lack of effective psychological resources or regulatory mechanisms may be associated with greater susceptibility to negative emotional experiences and functional impairments (Ingram and Luxton, 2005). Within this framework, ACEs are conceptualized as significant external stressors. Rumination reflects cognitive vulnerability, while PR represents internal psychological resources. In addition, PCEs are considered a protective moderator that may attenuate negative associations related to early-life risks. This theoretical integration provides a foundation for testing a putative sequential indirect association and the moderation mechanism examined in the current study.

### Adverse childhood experiences and mental health

ACEs encompass a range of severe negative events encountered during early development, including emotional neglect, physical abuse, family conflict, parental divorce, and caregiver mental illness (Curtis et al., 2025). As a prevalent source of early-life stress, ACEs have been associated with long-term differences in individuals' emotional regulation, self-concept, and interpersonal functioning. Extensive studies has demonstrated strong links between ACEs and various psychological problems in adolescence, including depression, anxiety, hostility, and self-injurious behavior (Cheng et al., 2025; Lee et al., 2022; Ye et al., 2024). Ye et al. (2024) emphasized that childhood is a critical stage for the formation of self-concept, and coping abilities. Prolonged exposure to high-stress or unsafe family environments during this stage may be associated with impaired emotional functioning, biased cognitive processing, and diminished psychological security. Children who undergo neglect or emotional deprivation tend to exhibit negative attributional styles and low self-esteem, which, in turn, may weaken their capacity to manage daily stressors and interpersonal setbacks (Feiler et al., 2023). Furthermore, research by Gautam et al. (2024) demonstrated that ACEs are associated with a greater likelihood of internalizing problems, and are closely related to externalizing behaviors, including aggression and impulsivity. These patterns may be linked to less healthy psychological functioning. As typical external stressors in the SVM, ACEs may co-occur with individual psychological vulnerabilities in ways that are associated with impaired MH (Ingram and Luxton, 2005). Taken together, prior evidence suggests that ACEs represent an important early-life risk correlate of adolescent MH.

### Indirect association involving rumination

Rumination is a maladaptive cognitive processing style defined by repetitive and passive attention to negative emotions and their causes following adverse events (Hu Y. et al., 2024). It has been linked to a longer duration of negative affect, impairs emotional regulation capacity, and reduced use of adaptive strategies (Li et al., 2023). According to Li et al. (2024), rumination is associated with negative self-evaluations and feelings of helplessness, thereby potentially undermining self-esteem and coping efficacy. Individuals with a high tendency to ruminate over time may experience emotional exhaustion and social withdrawal, which is associated with a greater likelihood of MH (Wu et al., 2024). Thus, rumination can be considered a potential psychological risk factor under conditions of stress.

ACEs have been identified as important correlates of ruminative thinking. Childhood exposure to emotional neglect, cold responses, or repeated invalidation has been associated with negative attributional styles and self-deprecating tendencies, increasing the likelihood of engaging in repetitive negative thinking when encountering setbacks (Kurtoǧlu et al., 2024). Such early adversity has also been linked to lower capacity for positive emotional reappraisal, making individuals tend to report ruminative responses to stress (Fox et al., 2024; Fu et al., 2024). Wang et al. (2023) similarly found that adolescents who had been exposed to early-life adversity tended to exhibit high levels of rumination, characterized by heightened reactivity, frequent internal conflict, and difficulty terminating negative thought cycles. Taken together, these findings suggest that rumination may be an important cognitive process involved in the association between ACEs and adolescent MH.

### Indirect association involving psychological resilience

PR denotes an individual's capacity to maintain psychological stability, adapt positively, and even grow when confronted with stress, challenges, or adversity (Yam et al., 2024). As a crucial internal protective factor, PR is associated with efficient emotion regulation, preserved self-esteem, and stronger problem-solving abilities, thereby potentially attenuating the detrimental associations between external stress and MH (Yang et al., 2024). Luo et al. (2025) reported that adolescents demonstrating stronger PR were more successful in maintaining emotional stability and adopting constructive coping strategies when facing academic, interpersonal, and familial stress. Moreover, PR is related to one's ability to mobilize social support and develop emotional connections with others, which may help reduce negative emotions and feelings of isolation (Lin et al., 2024). For these reasons, PR is widely regarded as a core psychological asset associated with adolescent MH and adaptive development.

ACEs are recognized as significant risk correlates of lower PR. Research has shown that children lacking positive interactions and secure attachments during early life are more likely to exhibit emotion regulation difficulties and avoidant coping tendencies, which may be associated with weaker PR (Elmore et al., 2020). In addition, such early adversity is often linked to a sense of helplessness and self-deprecation, potentially limiting the individual's ability to activate internal coping resources when facing future challenges. Individuals with these experiences may therefore report more withdrawal or breakdown reactions when exposed to adversity (Lackova Rebicova et al., 2021). Furthermore, ACEs have been associated with disruptions in emotional and cognitive recovery systems, which may correspond to lower stress adaptation and PR (Hu H. et al., 2024; Zhu et al., 2023). Taken together, these findings indicate that PR may represent an important psychological resource involved in the association between ACEs and adolescent MH.

### The sequential indirect association of rumination and psychological resilience

Prior research has shown that a strong tendency to ruminate is associated with poorer capacity to manage stress and regain emotional balance (Boucher et al., 2024; Yu and Liu, 2025). This association can be further understood within the SVM, which emphasizes that psychological maladjustment is related to the interplay among external stressors, individual vulnerabilities, and insufficient coping resources (Zubin and Spring, 1977). In the present study, rumination is conceptualized as a cognitive vulnerability that may be more proximal to stress-related negative cognitive processing, whereas PR reflects psychological resources associated with resilient adaptation. Persistent negative thinking is associated with emotional fluctuations and may interfere with individuals' ability to positively reframe problems and integrate available resources (Mansueto et al., 2022; Zagaria et al., 2023). This pattern may be linked to poorer self-regulation and adaptive functioning. Yu et al. (2024) noted that adolescents with a ruminative style tend to report cycles of negative affect when encountering adversity. Such cycles are associated with poorer emotional recovery and psychological repair processes, as well as lower flexibility and PR under stress (Ji, 2024). Therefore, from the perspective of the SVM, rumination may precede lower PR because it reflects a vulnerability process through which stress-related negative cognition may weaken the recovery, reappraisal, and coping-resource mobilization processes that support resilience. This reasoning provides a basis for considering rumination and PR as sequentially linked processes in the association between ACEs and adolescent MH.

### The moderating role of positive childhood experiences

Although ACEs are widely recognized as important risk correlates of poorer MH, not all individuals exposed to adversity report severe psychological problems. This variation suggests the presence of potential protective factors during development that may attenuate the negative associations related to such experiences (Almeida et al., 2024). PCEs, as a critical psychological protective resource, refer to emotionally supportive, affirming, and securely attached interactions, as well as positive social engagements that occur during early life (Şanli et al., 2024). Recent systematic review evidence has further emphasized the beneficial role of PCEs in children and adolescents, suggesting that positive early experiences may contribute to better psychological adjustment and may help counterbalance the negative associations linked to childhood adversity (Sousa et al., 2025). When adolescents have access to a certain level of PCEs, even in the context of adversity, they are more likely to develop basic trust, emotional security, and adaptive coping resources, which may support resilience and weaken the negative association between adversity and MH (Almeida et al., 2024; Deng et al., 2023). Van Doorn et al. (2024) emphasized that individuals who receive emotional support or experience positive social interactions while simultaneously encountering neglect or family conflict tend to show more stable emotional regulation patterns and positive social cognition. These capacities, in turn, may be associated with reduced psychological impairment in the context of ACEs (Bhargav and Swords, 2024). PCEs may buffer adolescents from the negative associations of ACEs, as previous research has shown that PCEs moderated the impact of ACEs on adolescent depression and anxiety (Qu et al., 2022). By attenuating vulnerability-related associations, PCEs may help preserve youth MH stability, particularly among those exposed to ACEs (Samji et al., 2024). Taken together, prior findings suggest that PCEs may function as a protective moderator in the associations of ACEs with adolescent MH and related psychological processes.

### The current study

Building on the SVM and prior empirical findings, the present study aimed to examine the association between ACEs and adolescent MH and to further clarify the psychological mechanisms and boundary conditions underlying this association. Specifically, this study tested whether rumination and PR were involved in independent and sequential indirect associations between ACEs and MH. In addition, the study examined whether PCEs moderated the associations of ACEs with rumination, PR, and MH. The following hypotheses were proposed:

H1: ACEs are significantly negatively associated with adolescent MH.
H2: Rumination is involved in a significant indirect association between ACEs and MH.
H3: PR is involved in a significant indirect association between ACEs and MH.
H4: Rumination and PR are involved in a significant sequential indirect association between ACEs and MH.
H5: PCEs moderate the association between ACEs and MH.
H6: PCEs moderate the association between ACEs and rumination.
H7: PCEs moderate the association between ACEs and PR.

**Figure 1:**
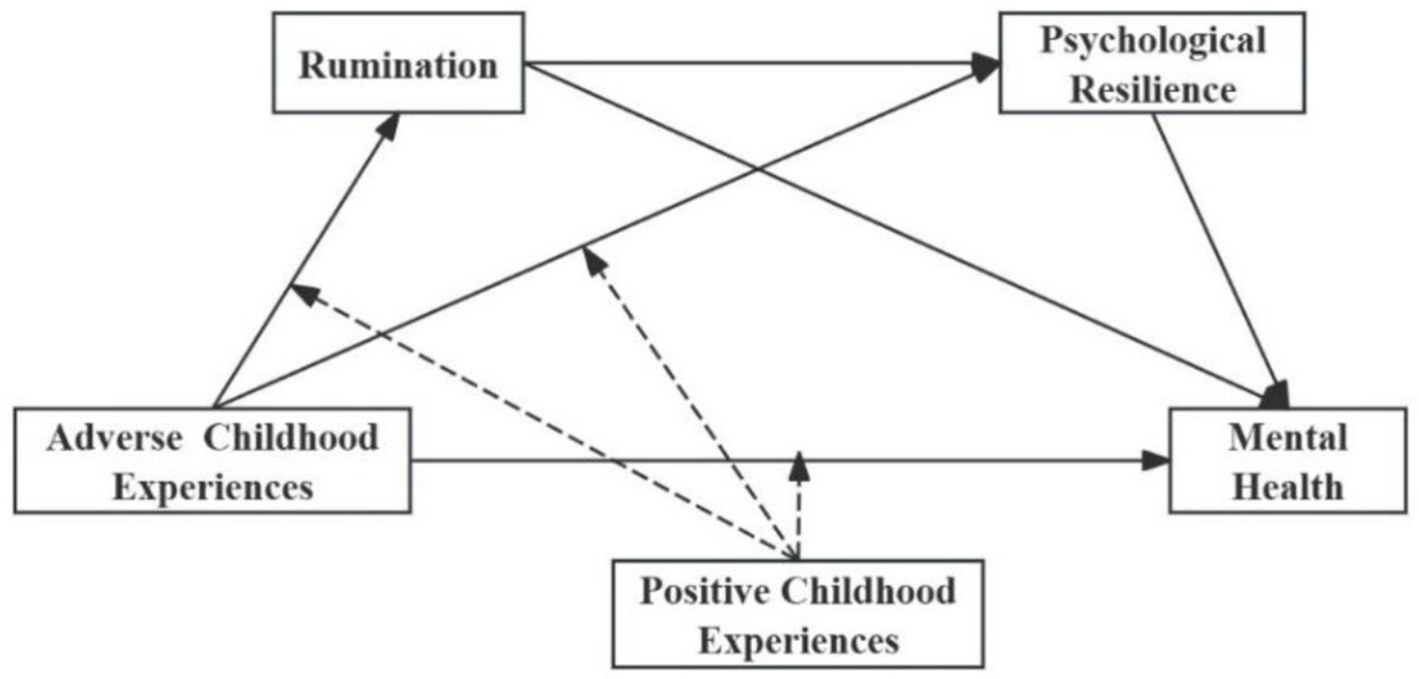
Theoretical hypothesized model.

The proposed theoretical model is illustrated in Figure 1.

## Methods

### Participants

This study collected data from three secondary schools in China between May and June 2025. The parents or legal guardians of the participants provided written informed consent, and the teenage participants gave their assent before taking part. Students were asked to use an online survey tool to fill out the questionnaire. Ethical approval for the study was granted by the Academic Committee of Baize University, covering all procedures related to participant recruitment, data collection, and data analysis. Every procedure adhered to the Declaration of Helsinki's ethical guidelines for human research. Based on a projected attrition rate of approximately 20% (Kline, 2018), a minimum sample size of 624 participants was deemed necessary. 800 samples were administered, with 756 valid responses received, yielding a response rate of 94.5%. After excluding responses with missing data or signs of careless answering, 700 valid questionnaires were included for analysis. The final sample consisted of 384 male (54.86%) and 316 female (45.14%) (Table 1).

**Table 1:** Demographic characteristics.

Variables	Category	Frequency	Percentage (%)
Gender	Male	384	54.86
	Female	316	45.14
Grade	Junior high school	423	60.43
	Senior high school	277	39.57
Household registration	Urban	409	58.43
	Rural	291	41.57
Only child	Yes	407	58.14
	No	293	41.86

### Measures

For both ACEs and PCEs, summed scores were computed to represent cumulative exposure to adverse or positive experiences. Because ACEs consist of checklists of distinct experiences rather than unidimensional constructs, internal consistency coefficients such as Cronbach's α are typically not reported. Psychometric evaluations have supported the structural validity of these measures in Chinese samples (Mi et al., 2023; Zhan et al., 2021).

### Adverse childhood experiences

ACEs were assessed using the Revised Adverse Childhood Experiences Questionnaire (ACEQ-R) developed by Peng and Ishak (2025). This scale has been adapted into Chinese and validated for use among Chinese adolescent populations (Jiang et al., 2022). The ACEQ-R comprises 10 binary items (0 = no, 1 = yes), covering domains including physical abuse, emotional abuse, physical neglect, and household dysfunction. The total score spans from 0 to 10, with higher scores reflecting greater experience of ACEs.

### Positive childhood experiences

PCEs were assessed using the Chinese version of the Benevolent Childhood Experiences Scale (BCEs) developed by Deng et al. (2023). The BCEs consists of 10 dichotomous items (0 = no, 1 = yes) and assesses three dimensions: perceived relational and internal safety, positive and predictable quality of life, and interpersonal support. The total scores span from 0 to 10, with higher scores reflecting a greater level of PCEs, Cronbach's alpha = 0.707.

### Rumination

Rumination was assessed using the Rumination Response Scale (RPS) adapted by Li et al. (2022) for Chinese adolescents. The RPS is a unidimensional scale comprises 10 items, each rated on a 4-point Likert scale, with higher average scores reflecting a greater tendency toward ruminative thinking. Previous studies have demonstrated that the 10-item RPS has good psychometric properties among Chinese adolescents (Gong et al., 2019). In the present study, Cronbach's alpha = 0.728.

### Psychological resilience

PR was assessed using the 10-item version of the Connor-Davidson Resilience Scale (CD-RISC-10) adapted by Mei et al. (2022) for Chinese adolescents. The CD-RISC-10 is a unidimensional scale comprising 10 items rated on a 5-point Likert scale, with higher scores reflecting higher levels of PR. The Chinese version of the CD-RISC-10 has been shown to be both reliable and valid (Ye et al., 2017). In the present study, Cronbach's alpha = 0.755.

### Mental health

MH was assessed using the Chinese version of the 12-item General Health Questionnaire (GHQ-12) adapted by Wang et al. (2022). The GHQ-12 comprises 12 items measuring three dimensions: depression/anxiety, social dysfunction, and loss of confidence. For example, one item is “I have felt unhappy and depressed.” Because the GHQ-12 is commonly used as a measure of psychological distress, with higher raw scores generally indicating poorer MH (Goldberg and Williams, 1988), the GHQ-12 items were reverse-coded before data analysis in the present study to construct a positively oriented MH indicator (Cheng et al., 2024; Simpson et al., 2023). Each item is rated on a 4-point Likert scale, with higher scores indicating better MH. In the present study, Cronbach's alpha = 0.733.

### Data analysis

The data were analyzed using SPSS and the PROCESS macro developed by Hayes (2012). The distributional features of the primary variables and the bivariate relationships between ACEs, PCEs, rumination, PR, and MH were first investigated using descriptive statistics and Pearson correlation analysis. To test the sequential mediation model, PROCESS Model 6 was used. Gender, grade, household registration, and only-child status were included as covariates to control for potential sociodemographic influences. These covariates were selected because adolescent MH and related psychological processes may vary according to gender, developmental stage, residential background, and family structure. Indirect effects were examined using a bootstrap procedure with 5,000 resamples. Bias-corrected 95% confidence intervals were calculated. To test the moderating effects, this study used PROCESS Model 85 and included the interaction terms between ACEs and PCEs. Significant interaction effects were further interpreted using simple slope analyses.

## Results

### Descriptive statistics

To ensure the robustness of statistical inferences, the normality of the data was assessed through skewness and kurtosis measures. As suggested by Kline (2005), acceptable markers of a normal distribution are absolute skewness values less than 2 and absolute kurtosis values less than 7. The data in this investigation roughly followed a normal distribution (Table 2).

**Table 2:** Descriptive statistics of main variables.

Variables	*N*	M ±SD	Min	Max	SK	Kur
ACEs	700	0.620 ± 0.907	0	10	1.804	3.934
PCEs	700	6.204 ± 2.460	0	10	−0.560	−0.361
Rumination	700	2.648 ± 0.383	1	4	−0.517	3.070
PR	700	3.315 ± 0.532	1	5	−1.560	4.890
MH	700	2.670 ± 0.382	1	4	−1.818	5.479

### Common method bias (CMB) test

CMB was assessed using Harman's single-factor test. First unrotated factor explained approximately 16.73% of the total variance, substantially lower than the critical threshold of 40% (Podsakoff et al., 2003), suggesting that CMB was not a major concern. However, we acknowledge that relying solely on this test is insufficient to fully rule out CMB. Given that all variables were collected via self-report at a single time point, the potential influence of CMB cannot be completely excluded.

### Correlation analysis

As shown in Table 3, ACEs were positively correlated with rumination, and negatively correlated with PR and MH. In addition, rumination was negatively correlated with PR and MH. Furthermore, PR was positively correlated with MH.

**Table 3:** Pearson correlations among variables.

Variable	ACEs	Rumination	PR	MH
ACEs	1	
Rumination	0.319^***^	1
PR	−0.534^***^	−0.724^***^	1	
MH	−0.524^***^	−0.695^***^	0.780^***^	1

^***^p < 0.001.

### Collinearity diagnostics

Multicollinearity was not an issue in this investigation because all variables had tolerance values greater than 0.1 and VIF below the suggested threshold of 3.3 (Hair et al., 2019) (Table 4).

**Table 4:** Collinearity diagnostics of independent variables.

Variables	Tolerance	VIF
ACEs	0.705	1.419
Rumination	0.469	2.132
PR	0.373	2.680

VIF, variance inflation factors.

### Indirect association analysis

**Figure 2:**
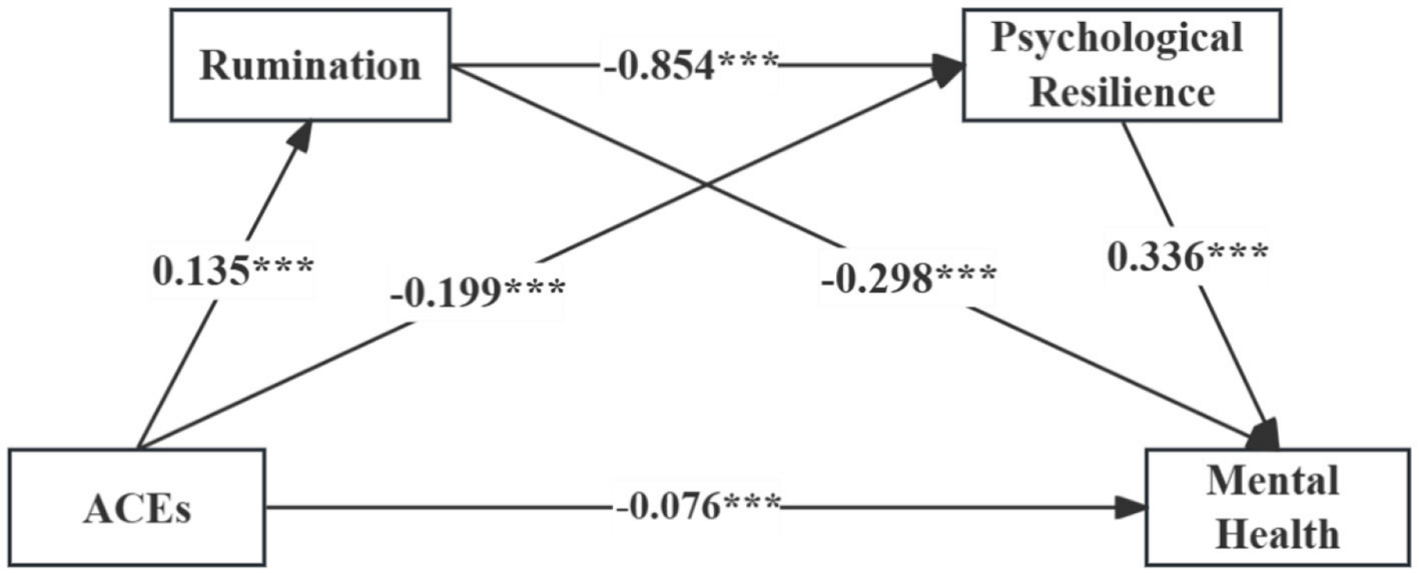
Standardized path coefficients for the indirect association model.

After controlling for gender, grade, household registration, and only-child status, we conducted the mediation analysis to examine the indirect effects of rumination and PR in the relationship between ACEs and MH. As shown in Table 5 and Figure 2, ACEs were significantly negatively associated with PR and MH, and significantly positively associated with rumination. Rumination was significantly negatively associated with both PR and MH. In addition, PR was significantly positively associated with MH.

**Table 5:** Regression results for the indirect association model.

Outcome variable	Predictor variable	β	SE	T	CI (95%)		*R* ^2^	*F*
					LLCL	ULCI		
Rumination	ACEs	0.135	0.015	8.864^***^	0.105	0.164	0.105	16.294
	Gender	0.001	0.028	0.045	−0.054	0.057		
	Grade	0.043	0.030	1.438	−0.016	0.102		
	Household registration	−0.029	0.029	−0.995	−0.086	0.028		
	Only child	−0.006	0.028	−0.229	−0.062	0.049		
PR	ACEs	−0.199	0.014	−13.883^***^	−0.227	−0.171	0.629	196.119
	Rumination	−0.854	0.034	−25.123^***^	−0.921	−0.787		
	Gender	−0.024	0.025	−0.955	−0.074	0.026		
	Grade	−0.045	0.027	−1.679	−0.098	0.008		
	Household registration	< 0.001	0.026	< 0.001	−0.051	0.051		
	Only child	0.013	0.025	0.495	−0.037	0.062		
MH	ACEs	−0.076	0.011	−6.930^***^	−0.098	−0.055	0.669	199.638
	Rumination	−0.298	0.032	−9.332^***^	−0.360	−0.235		
	PR	0.336	0.026	13.036^***^	0.286	0.387		
	Gender	0.021	0.017	1.230	−0.013	0.055		
	Grade	−0.014	0.018	−0.760	−0.050	0.022		
	Household registration	−0.023	0.018	−1.288	−0.058	0.012		
	Only child	0.009	0.017	0.518	−0.025	0.043		

^***^p < 0.001.

Table 6 presents the results of the indirect association analysis. The findings indicate that the indirect association via rumination was significant. In addition, the indirect association via PR was also significant. Furthermore, a sequential indirect association was observed, in which rumination and PR were jointly involved in the association between ACEs and MH.

**Table 6:** Indirect association analysis.

Effect	Path	β	SE	CI (95%)		Percentage	Result
			LLCI	ULCI		
Total effect	ACE → MH	−0.222	0.014	−0.249	−0.195	100%	Support
Direct effect	ACE → MH	−0.076	0.011	−0.098	−0.055	34.2%	Support
Indirect effect	ACE → Rumination → MH	−0.040	0.007	−0.054	−0.026	18.0%	Support
	ACE → PR → MH	−0.067	0.008	−0.083	−0.051	30.2%	Support
	ACE → Rumination → PR → MH	−0.039	0.008	−0.054	−0.023	17.6%	Support

### Robustness checks

To examine the robustness of the findings, the main analyses were repeated without covariates. The results were substantively consistent with those of the primary models. Specifically, the indirect associations via rumination and PR, as well as the sequential indirect association through rumination and PR, remained significant (Table 7). These findings suggest that the main results were robust across model specifications.

**Table 7:** Robustness check of the indirect association analysis without covariates.

Effect	Path	β	SE	CI (95%)		Percentage (%)	Result
				LLCI	ULCI		
Total effect	ACE → MH	−0.221	0.014	−0.248	−0.194	100	Support
Direct effect	ACE → MH	−0.075	0.011	−0.097	−0.054	33.9	Support
Indirect effect	ACE → Rumination → MH	−0.040	0.007	−0.054	−0.025	18.1	Support
	ACE → PR → MH	−0.067	0.008	−0.083	−0.051	30.3	Support
	ACE → Rumination → PR → MH	−0.039	0.008	−0.054	−0.023	17.7	Support

### Moderation analysis

As shown in Table 8, PCEs significantly moderated the associations between ACEs and rumination, PR, and MH. To further interpret these interactions, simple slope analyses were conducted at low (M – 1SD = 3.744), medium (M = 6.204), and high (M + 1SD = 8.664) levels of PCEs. As shown in Table 9, ACEs were positively associated with rumination at low and medium levels of PCEs, but this association was nonsignificant at high levels of PCEs. ACEs were negatively associated with PR across all three levels of PCEs, although the magnitude of this association became weaker as PCEs increased. In addition, ACEs were negatively associated with MH at low and medium levels of PCEs, but this association became non-significant at high levels of PCEs. Taken together, these results indicate that PCEs attenuated the associations of ACEs with higher rumination, lower PR, and poorer MH, suggesting a protective buffering pattern.

**Table 8:** Moderating associations of PCEs.

Outcome variable	Predictor variable	β	SE	CI (95%)		Result
				LLCI	ULCI	
MH	ACEs × PCEs	0.023^***^	0.003	0.017	0.029	Support
Rumination	ACEs × PCEs	−0.035^***^	0.004	−0.043	−0.026	Support
PR	ACEs × PCEs	0.034^***^	0.004	0.026	0.043	Support

^***^p < 0.001.

**Table 9:** Conditional effects of ACEs on outcomes at different levels of PCEs.

Outcome variable	PCEs level	PCEs value	Effect	SE	*t*	*p*
Rumination	Low, M – 1SD	3.744	0.153	0.015	10.474	< 0.001
Rumination	Mean	6.204	0.067	0.015	4.533	< 0.001
Rumination	High, M + 1SD	8.664	−0.018	0.021	−0.857	0.392
PR	Low, M – 1SD	3.744	−0.247	0.015	−16.425	< 0.001
PR	Mean	6.204	−0.162	0.014	−11.241	< 0.001
PR	High, M + 1SD	8.664	−0.078	0.020	−3.810	< 0.001
MH	Low, M – 1SD	3.744	−0.118	0.012	−9.912	< 0.001
MH	Mean	6.204	−0.062	0.011	−5.853	< 0.001
MH	High, M + 1SD	8.664	−0.005	0.014	−0.392	0.695

## Discussion

ACEs showed a significant negative association with adolescents' MH, thereby supporting H1. This interpretation is based on the reverse-scored GHQ-12 composite used in the present study, in which higher MH scores represent better MH rather than greater psychological distress. Thus, the negative association indicates that adolescents reporting more ACEs tended to report poorer MH. According to the SVM (Zubin and Spring, 1977), ACEs can be conceptualized as typical early-life stressors that may be linked to persistent emotional responses and difficulties in psychological adaptation. In the context of Chinese culture, the emotional foundation provided by the family plays a particularly important role in adolescent development (Wan et al., 2022). When individuals experience emotional deprivation or family dysfunction during childhood, their emotional regulation systems and basic trust structures may be compromised and associated with poorer overall MH (Gao et al., 2022). Therefore, the current findings further support ACEs as a critical risk correlate of adolescent MH. These results underscore the importance of considering early family environments in both school-based psychological education and clinical intervention, particularly in the design of early risk identification and prevention systems.

Rumination showed a significant indirect association in the relationship between ACEs and adolescent MH, thereby supporting H2. In this study, the indirect association through rumination accounted for 18.0% of the total association. Specifically, ACEs were associated with higher levels of rumination, suggesting that adolescents with more ACEs may tend to report a habitual tendency to think repetitively about negative events. Rumination, in turn, was associated with poorer psychological flexibility in coping with stress and poorer MH (Fu et al., 2024; Wu et al., 2024). This study offers cross-cultural support for the applicability of this indirect association model within a Chinese adolescent population. It also broadens the theoretical scope of the model by emphasizing the role of cognitive processing patterns under conditions of developmental stress. These findings offer novel empirical support for understanding rumination as a putative cognitive mechanism that may help explain the association between ACEs and psychological difficulties, and provide a theoretical foundation for interventions targeting cognitive regulation to improve adolescent MH.

PR showed a significant indirect association in the relationship between ACEs and adolescent MH, thereby supporting H3. In the present study, the indirect association involving PR accounted for 30.2% of the total association, indicating that PR was an important variable in the risk pattern. Specifically, ACEs were associated with lower PR, suggesting that adolescents with more adverse experiences may report weaker feelings of security and coping efficacy (Morgan et al., 2022). Adolescents with lower PR tend to exhibit withdrawal responses and emotional breakdowns when confronted with challenges, and they tend to lack the recovery capacity and adaptability needed to buffer psychological distress (Uslu and Ime, 2025). By identifying PR as a putative mediating variable, this study highlights its importance as a protective psychological resource in risk adaptation. The findings provide new empirical evidence for understanding psychological vulnerability in adolescence and underscore the value of resilience-focused interventions for promoting youth MH.

Rumination and PR showed a significant sequential indirect association between ACEs and adolescent MH, supporting H4. Rumination is associated with intensified negative emotional experiences and with lower PR, possibly through difficulties in maintaining emotional balance and recovering functioning under stress (Lian et al., 2021). In this study, the sequential indirect association accounted for 17.6% of the total association, indicating that rumination and PR together formed a cognitive-resource association pattern linking ACEs to MH outcomes. This finding expands the explanatory scope of the SVM (Zubin and Spring, 1977). Unlike traditional interpretations that emphasize the interaction between stressors and individual vulnerabilities, the present study identifies a novel cognitive-resource pattern through which early stress may be statistically associated with adolescent MH. This integrative perspective enriches our understanding of how cognitive processing (e.g., rumination) and internal psychological resources (e.g., PR) are related to developmental risk trajectories. Moreover, the findings offer practical insights for multilevel intervention strategies, suggesting that future prevention and intervention efforts may benefit from targeting both cognitive regulation and PR enhancement to reduce the long-term psychological risks associated with ACEs.

PCEs significantly moderated the association between ACEs and adolescent MH, supporting H5. This finding supports the conceptualization of PCEs as an external protective factor, and aligns with Almeida et al. (2024), indicating that even in the context of adversity, individuals who receive emotional support, secure attachment, or affirmative responses during development tend to report psychological adaptability and cognitive flexibility that buffer risk-related associations. PCEs may be associated with adolescents' basic perception of trust, self-worth, and emotional security, helping to attenuate maladaptive cognitive and emotional responses to stressors (Bhargav and Swords, 2024). The moderating pattern identified in this study not only supports the hypothesized model but also provides empirical evidence for understanding why individuals exposed to similar early-life adversities may show different psychological developmental patterns.

PCEs significantly moderated the association between ACEs and rumination, supporting H6. In particular, adolescents who reported more PCEs showed a weaker association between ACEs and the tendency to ruminate. This finding aligns with the results of Qu et al. (2022), which suggest that PCEs may be associated with emotional security, self-acceptance, and optimistic expectations toward the environment, thereby supporting more flexible coping strategies and a lower likelihood of engaging in repetitive trauma-related thoughts or self-blame. Furthermore, PCEs may be linked to more constructive interpretations of adversity and a greater capacity for meaning-making, which in turn may correspond to less cognitive fixation on negative events and weaker ruminative thinking patterns (Zhang et al., 2021). This moderating pattern not only supports the hypothesized model but also enhances theoretical insight into the development and buffering of cognitive vulnerability.

PCEs significantly moderated the association between ACEs and PR, supporting H7. Liu et al. (2026) indicate that although ACEs may be present, individuals who receive emotional connection, affirming responses, or secure attachment may still report stronger adaptive capacities and self-repair mechanisms. PCEs may be associated with trust and perceived control, thereby helping individuals form more constructive coping styles and self-appraisals (Deng et al., 2023). As a result, the lower PR typically associated with ACEs may be attenuated among adolescents with more PCEs. Taken together, these results highlight a protective moderating pattern whereby PCEs weaken the negative association between ACEs and PR, which may be consistent with reduced risk accumulation along the stress-related pathway.

The present findings clarify the role of PCEs as primarily protective factors in the associations between ACEs and adolescent MH. Theoretically, promotive factors are expected to show beneficial effects regardless of risk exposure, whereas protective factors specifically attenuate the negative impact of risk factors (Samji et al., 2024; Wolke et al., 2025). In the present study, the significant ACEs × PCEs interactions, together with the simple slope results, showed that the associations of ACEs with higher rumination, lower PR, and poorer MH became weaker as PCEs increased. In particular, the associations of ACEs with rumination and MH were no longer significant at high levels of PCEs. Therefore, PCEs appeared to operate mainly through a protective buffering mechanism rather than merely through a general promotive or compensatory effect.

## Implications

### Theoretical implications

The findings offer several important theoretical contributions that extend the application of this model within the field of adolescent developmental psychology. First, the study showed that rumination and PR were involved in significant indirect associations between ACEs and MH. This highlights the potential role of cognitive processing patterns and internal psychological resources in accounting for the association between early adversity and later psychological outcomes, thereby deepening the theoretical understanding of how risk and vulnerability factors may be interrelated. Second, adopting an integrative perspective that considers both risk and protective factors, the study found that PCEs attenuated the negative association between ACEs and MH and also moderated the associations of ACEs with rumination and PR. These findings expand the theoretical boundary of the SVM by introducing external protective resources as moderators operating across multiple levels and pathways. This offers a new conceptual lens for understanding how adolescents adapt to ACEs through complex, multi-level association patterns. Finally, by focusing on a Chinese adolescent sample, the study provides a culturally grounded theoretical contribution. It adds to the theoretical modeling of adversity adaption mechanisms in developmental psychology and provides empirical evidence for links between early family events and psychological development patterns in a non-Western setting.

### Practical implications

The findings of this study have important practical implications for the development of effective intervention strategies to promote adolescent MH. First, at the family level, it is crucial to reduce the risk of ACEs. Parents should be made more aware of the potential harm associated with emotional neglect, verbal abuse, and family dysfunction. Parenting education programs and psychological counseling services can be implemented to improve caregivers' emotional regulation skills and foster positive parent-child interactions, thereby supporting a safer and more nurturing environment for child development. Second, at the school level, attention should be given to identifying and addressing students' tendencies toward rumination and their levels of PR. Schools can establish regular psychological screening mechanisms to detect students with high ruminative tendencies and provide targeted cognitive-behavioral interventions (e.g., thought-stopping training, positive self-talk exercises) that may help them break negative thinking cycles. Moreover, programs aimed at PR building (e.g., emotion regulation workshops, problem-solving skills training, and group counseling), should be widely implemented to strengthen students' ability to manage stress and adversity. Third, at the community and policy level, efforts should be made to promote early childhood development support services and increase access to PCEs. Initiatives such as family reading days, emotional support groups, and developmental camps can offer children enriched opportunities for positive engagement. Policymakers should also invest in family support programs, including parenting helplines and family conflict mediation platforms, to build a comprehensive, multilayered intervention network that both prevents ACEs and strengthens the protective mechanisms that support adolescent MH.

### Limitations and future directions

First, the study adopted a cross-sectional design, which allowed for the identification of structural relationships among variables but did not permit conclusions regarding temporal ordering or causality. Future research may consider using longitudinal tracking or experimental intervention designs to examine how childhood experiences dynamically affect psychological mechanisms and health development over time, thereby enhancing the validity of causal inferences. Second, the data were mainly obtained through adolescent self-report measures, and the retrospective assessment of ACEs and PCEs may be subject to recall bias. Although procedural controls were implemented to reduce potential biases, relying exclusively on self-report data collected at a single time point may introduce CMB. While preliminary assessments such as Harman's single-factor test can provide initial indications, they are insufficient to fully rule out CMB. Therefore, the results should be interpreted with caution, and future studies are encouraged to incorporate multiple sources of information to improve reliability and ecological validity. Third, gender-based analyses were not conducted in this study. Although gender was included as a covariate in the main models, potential differences in the associations among ACEs, PCEs, rumination, PR, and MH across male and female adolescents remain unexplored. Future research could explicitly examine gender differences to provide a more nuanced understanding of these relationships. Fourth, the sample consisted of school-attending adolescents from a Chinese cultural context, where family structure and social support systems may differ from those in other cultures. To investigate the applicability and stability of the suggested model in other sociocultural contexts, future research could undertake cross-cultural comparison studies. This would strengthen the generalizability of the results and expand the theoretical understanding of how individuals adapt to early adversity in diverse cultural environments.

## Conclusion

Grounded in the SVM, this study tested a serial multiple indirect association model to systematically explore the associations linking ACEs to MH among adolescents. Rumination and PR were examined as variables involved in indirect associations, while PCEs were examined as a moderator. ACEs were negatively associated with adolescents' MH. This association was evident not only in the direct pathway but also through independent indirect associations via rumination and PR, as well as a sequential indirect association involving rumination and PR. In addition, PCEs moderated the associations between ACEs and rumination, PR, and MH. Overall, this study deepens the understanding of how childhood experiences relate to adolescent MH and offers multi-path, multi-level theoretical and practical implications for psychological intervention.

## Data Availability

The datasets presented in this study can be found in online repositories. The names of the repository/repositories and accession number(s) can be found in the article/supplementary material.
